# Special Issue “Invasion and Metastasis in Brain Cancer”

**DOI:** 10.3390/ijms27031555

**Published:** 2026-02-05

**Authors:** Aleksandra Glogowska, Saeid Ghavami

**Affiliations:** 1 Department of Human Anatomy and Cell Science, Faculty of Health Science, College of Medicine, University of Manitoba, Winnipeg, MB R3E 0T4, Canada; 2 Paul Albrechtsen Research Institute Cancer Care Manitoba, Winnipeg, MB R3E 0V9, Canada; 3 Children Hospital Research Institute of Manitoba, Winnipeg, MB R3E 0Z3, Canada; 4 Faculty of Medicine, Akademia Śląska, Rolna 43, 40-555 Katowice, Poland

Brain tumors, whether primary gliomas or metastases from breast, lung, melanoma, or other systemic malignancies, share a unique biological feature: the ability to infiltrate brain parenchyma and adapt to the unique microenvironment of the central nervous system [1,2,3,4,5,6]. This invasive capacity renders complete surgical resection impossible and underlies recurrence and resistance to current therapies [7,8,9,10]. This Special Issue underlines that invasion is not a secondary phenomenon but a core driver of poor outcome, and that meaningful therapeutic progress will require mechanistic insights into how tumor-intrinsic programs intersect with microenvironmental cues to promote dissemination, survival, and treatment failure [8,11,12,13]. Advances in understanding extracellular matrix remodeling, cell–cell and cell–matrix interactions, metabolic rewiring, and bidirectional tumor–microenvironment signaling are therefore essential for biomarker discovery and the development of precision, mechanism-based therapies [14,15,16,17,18].

All contributions emphasize that the peritumoral brain zone is a biologically active and permissive niche rather than a passive boundary. Spatial and molecular profiling studies demonstrate that invasive margins harbor distinct lipidomic, metabolic, and regulatory programs that differ from tumor cores [19,20,21,22]. Ica et al. (contribution 1) identify region-specific ganglioside enrichment at astrocytoma margins using ion mobility mass spectrometry, reinforcing the concept that lipid remodeling contributes to migration and local adaptation [19]. These findings are consistent with broader metabolomic and miRNA studies showing that peritumoral regions support invasion through altered metabolism, immune modulation, and glial interactions [20,21,22]. Complementing this, De Fazio et al. (contribution 2) synthesize current knowledge on glioblastoma invasion, highlighting how cytoskeletal dynamics, integrin signaling, extracellular matrix remodeling, and neuronal and immune interactions converge to enable diffuse infiltration and therapy resistance [23,24]. Together, these studies emphasize that targeting invasion will require strategies that address both tumor-intrinsic heterogeneity and dynamic microenvironmental support.

The Special Issue further underscores that inflammatory and genetic regulators critically shape invasive and metastatic behavior. Jablonska et al. (contribution 3) reveal persistent STAT3 activation in post-radiation necrosis, highlighting how treatment-induced microenvironmental remodeling may promote immune suppression and recurrence [25,26]. At the genetic level, Boix De Jesus et al. (contribution 4) identify the Δ133p53β isoform as a consistent marker of brain metastatic competence across tumor types, linking it to AKT and MAPK activation and enhanced endothelial transmigration [27,28,29]. Similarly, UBE2C emerges as a multifunctional driver of proliferation, epithelial–mesenchymal transition, and motility in intracranial tumors (contribution 5) [30,31]. Finally, breast cancer brain metastasis exemplifies how tumors exploit blood–brain barrier signaling, vascular co-option, metabolic adaptation, and immune evasion to establish lethal secondary lesions (contribution 6) [32,33].

This volume communicates that invasion and brain colonization are dictated and controlled by tumor genetics and a specialized developed microenvironment. Effective therapies should therefore integrate molecular targeting with strategies that disrupt tumor–microenvironment crosstalk with tumor components. By diverging insights from spatial profiling, signaling cell biology approaches, and translational research, this Special Issue highlights actionable/targetable pathways and conceptual frameworks that can guide next-generation interventions aimed at limiting invasion, preventing recurrence, and improving outcomes for patients with primary and metastatic brain cancers [21,22,23,24,25,26,27,28,29,30,31,32,33].
